# Effect of respiration on Korotkoff sounds and oscillometric cuff pressure pulses during blood pressure measurement

**DOI:** 10.1007/s11517-014-1150-1

**Published:** 2014-03-26

**Authors:** Dingchang Zheng, Luigi Yuri Di Marco, Alan Murray

**Affiliations:** Cardiovascular Physics and Engineering Research Group, Institute of Cellular Medicine, Newcastle University, Newcastle, NE2 4HH UK

**Keywords:** Blood pressure measurement, Oscillometric pulses, Korotkoff sounds, Respiratory modulation

## Abstract

Blood pressure (BP) measurement accuracy depends on consistent changes in Korotkoff sounds (KorS) for manual measurement and oscillometric pulses for automated measurement, yet little is known about the direct effect of respiration on these physiological signals. The aim of this research was to quantitatively assess the modulation effect of respiration on Korotkoff sounds and oscillometric pulses. Systolic and diastolic blood pressures were measured manually from 30 healthy subjects (age 41 ± 12 years). Three static cuff pressure conditions were studied for two respiratory rates. Cuff pressure [with oscillometric pulses (OscP)], ECG, chest motion respiration [respiration signal (Resp), from magnetometer] and Korotkoff sounds (KorS, from digital stethoscope) were recorded twice for 20 s. The physiological data were evenly resampled. Respiratory frequency was calculated from Resp (f_R_), OscP (f_O_) and KorS (f_K_) from peak spectral frequency. There was no statistically significant difference between f_R_ and f_O_ or f_K_. Respiratory modulation was observed in all subjects. OscP amplitude modulation changed significantly between the two respiratory rates (*p* < 0.05) and between the three cuff pressures (*p* < 0.0001), and decreased significantly with decreasing cuff pressure (*p* < 0.05). The phase shift between Resp and modulation of OscP was statistically significant with respiratory rates (*p* < 0.05), but not with cuff pressures. It is accepted that BP in individuals is variable and that this relates to respiration; we now show that this respiration modulates oscillometric pulse and Korotkoff sound amplitudes from which BP is measured.

## Introduction

It is well accepted that respiration influences beat-by-beat blood pressure (BP) changes [6]. During the respiratory cycle, changes occur in the central venous pressure as a consequence of chest expansion and compression. During inspiration, the decrease in central venous pressure increases venous return and right atrial filling and at the same time reduces pulmonary venous flow to the left side of the heart, leading to reduced stroke volume and decreased mean systemic arterial pressure. During expiration, the opposite occurs.

The physiological interactions during respiration are complex. In 1952, Dornhorst et al. [6] documented the effect of respiration on direct BP and showed phase differences between them. Later, Saul et al. [13] extended these observations from invasive recordings of the radial arterial pressure with respiration at a constant mean respiratory rate, and Laude et al. [9] studied these phases at different respiratory frequencies. Many different techniques have been used to study the phase relationship between the inter-beat interval (or instantaneous heart rate) and the systolic blood pressure (SBP) recorded noninvasively with paced breathing. They include frequency domain cross-spectral analysis [2, 14, 17, 19], *wind*-*kessel* model [5], time domain cross-correlation [3, 9], mutual information analysis [20] and a numeric model of the closed loop regulation system [13].

There are currently two common noninvasive techniques (manual auscultatory and automated oscillometric) to determine BPs. Manual auscultatory technique is based on the auscultation of the Korotkoff sound, whereas automated measurement is almost always based on the oscillometric pulse waveform. BP measurement accuracy therefore depends on consistent changes in Korotkoff sounds for manual measurement and oscillometric pulses for automated measurement.

Although the exact mechanisms underlying the respiratory influence on BP measurement are not fully understood, it is likely that oscillometric pulses recorded from cuff pressure during BP measurement is influenced by respiration. Moreover, if respiration modulates the oscillometric pulses, this modulation could also affect the Korotkoff sounds associated with the blood pulse flowing through the brachial artery. One longstanding hypothesis on the genesis of Korotkoff sounds is that the sharp audible tapping sound is generated by the distension of the arterial wall caused by the changing transmural pressure gradient [4, 10, 16, 18]. It may therefore be speculated that if respiration modulates the pressure pulse, it might also influence the force deployed in opening the artery, which in turn may reflect on the amplitude of the Korotkoff sounds.

Our previous study has quantified the clinical importance of the effect of respiration on BP measurement. With regular deeper breathing, both SBP and diastolic blood pressure (DBP) changed significantly in comparison with normal condition: decreasing by 4.4 and 4.8 mmHg, respectively [21]. A major review in the Journal of the American Medical Association (JAMA) estimated that a 5-mmHg error either above or below the actual BP would result in 27 million Americans being exposed to unnecessary treatment or 21 million being denied treatment [8]. Therefore, any small BP changes caused by respiration are clinically important and worth further investigation.

Since the respiratory modulation of the oscillometric waveform and Korotkoff sound could influence the accuracy of BP measurement, it is therefore of clinical interest to quantify the influence of respiration on both signals, and further investigation could provide additional insight into BP measurement variability. However, to the best of our knowledge, no data have been reported on the direct effect of respiration on oscillometric pulses and Korotkoff sounds.

The aim of this research was to study such effects by quantitatively assessing: (1) the presence of a modulating effect of respiration on oscillometric pulse amplitude and Korotkoff sound amplitude; (2) different amplitude modulation of oscillometric pulses and Korotkoff sounds between different respiratory rates and cuff pressures; (3) the respiratory modulation of phase relationship between respiration and changes in the oscillometric pulses and Korotkoff sounds signals.

## Methods

### Subject data

Thirty healthy subjects were enrolled in the study (14 male, 16 female). Clinical information for those subjects is summarized in Table 1. The study was carried out in accordance with the Declaration of Helsinki (1989) of the World Medical Association and was approved by the locally appointed Ethics Committee. All subjects gave their written informed consent before participating.

**Table 1:** Summary clinical details for the 30 subjects participating in the study

	Mean ± SD
Age (years)	41 ± 12
Systolic blood pressure (mmHg)	116 ± 16
Mean arterial pressure (mmHg)	88 ± 11
Diastolic blood pressure (mmHg)	74 ± 10
Arm circumference (cm)	29 ± 3

Data are presented as mean ± SD

### Experimental protocol

For each subject, SBP and DBP were measured manually with the subject sitting quietly by a trained operator according to the recommendations of the European Hypertension Societies [11]. Physiological signals, including the cuff pressure, chest wall movement and Korotkoff sounds (KorS), were recorded for 20 s under three static cuff pressure conditions: high (H) SBP-10 mmHg, medium (M) (SBP + DBP)/2 and low (L) DBP + 10 mmHg. The order of three cuff pressures was randomized for each subject, and they were studied for two respiratory metronome rates): 0.20 Hz (12 breaths/min) and 0.30 Hz (18 breaths/min). These six recordings were then repeated after a 1-min rest period, giving a total of 12 recordings for each subject. Repeatability was assessed from the two repeat measurements, and average values from the two measurements were calculated for both SBP and DBP.

Breathing was paced using a visual metronome with equal inspiration and expiration periods. Subjects were instructed on how to follow the metronome and were given familiarization time before recordings started.

### Data acquisition

**Fig. 1:**
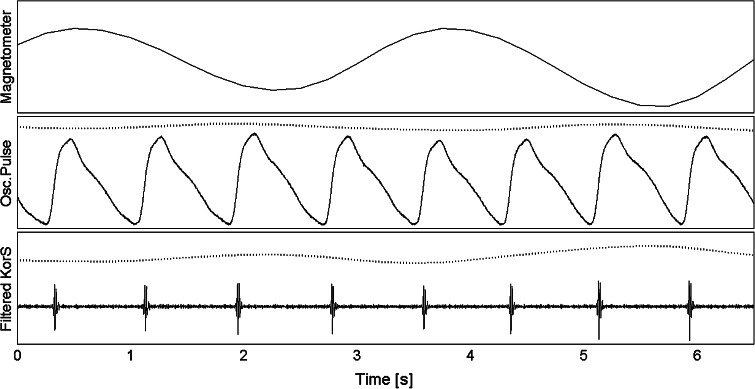
Examples of physiological data for two consecutive respiratory cycles from a subject breathing at 0.3 Hz (18 breaths/min). *Top panel* chest magnetometer signal. *Central panel* oscillometric pulse (*solid trace*) and estimated respiratory modulation (*dotted trace*, displaced for visibility). *Bottom panel* band-pass filtered KorS (*solid trace*) with estimated respiratory modulation (*dotted trace*). All *vertical scales* are in arbitrary units

The cuff pressure containing the oscillometric pulses (OscP) was recorded together with a single-lead (lead II) electrocardiogram (ECG). A chest magnetometer was used to record chest wall movement [7], and hence, a reference respiration signal (Resp) was obtained. A piezo-electric microphone was used to record KorS, with the bell-shaped stethoscope terminal connected to the microphone and placed on the antecubital fossa of the forearm in the position where the blood pulse was most audible with the cuff inflated at (SBP + DBP)/2. The stethoscope was secured to the arm with adhesive medical tape. All signals were recorded simultaneously, sampled at 2 kHz, 16-bit/sample and stored to a computer for off-line processing. Figure 1 shows the examples of Resp, OscP and KorS for two consecutive respiratory cycles from a subject breathing at 0.3 Hz.

### Signal processing

#### RR interval with different respiratory frequency

An established threshold-based QRS detection algorithm was used to detect the ECG R wave [12]. The mean RR interval across all detected heartbeats for each recording was calculated and used for the comparison between the two respiratory rates and between the three cuff pressures.

#### Oscillometric pulse amplitude

The foot of the oscillometric pulse was automatically identified in a fixed 200-ms window following the R wave. For each oscillometric pulse, the pulse amplitude (peak-to-nadir) was measured as the amplitude difference between the peak and the foot.

#### Korotkoff sound amplitude

The KorS signal was band-pass filtered (3 dB pass-band 59–1,000 Hz) to remove baseline noise. This band encompasses the frequency band of interest in KorS analysis used in [1]. For each pulse, the peak-to-nadir amplitude in the corresponding KorS was retained to generate the time series of KorS amplitude.

#### Estimation of respiratory rate from the time series of oscillometric and Korotkoff amplitudes

As the OscP and KorS amplitudes were separated by varying pulse intervals, they were evenly resampled at 4 Hz by cubic spline interpolation. This sample rate satisfies the Nyquist condition as the respiratory rate in resting conditions is generally contained in the range 0.1–0.5 Hz. This interpolation and resampling methods are commonly used in preliminary processing for the spectral analysis of the RR interval time series, which is also modulated by respiration (respiratory sinus arrhythmia) [15]. The magnetometer Resp was then also resampled at 4 Hz that was time aligned to the OscP and KorS amplitude data with the respiratory depth calculated from the peak-to-nadir of the respiratory waveform.

The respiratory modulation signals from Resp, OscP and KorS were then zero-meaned (mean value was subtracted from signals), detrended (1st order trend was removed) and zero-padded to 64 s. The respiratory frequency was calculated from Resp (f_R_), OscP (f_O_) and KorS (f_K_) as the peak frequency in the power spectral distribution estimated by the Welch periodogram using a Hamming window, between 0.1 and 0.5 Hz, with a resolution of 16 mHz.

The distributions of the frequency difference between Resp and that calculated from KorS amplitude (f_R_–f_K_), and between Resp and OscP amplitude (f_R_–f_O_) were calculated across all recordings. The presence of respiratory modulation was accepted when the modulated peak frequency in OscP or KorS amplitude was detected within the spectral resolution of 16 mHz. The histogram of their frequency differences was plotted with the bin width of 16 mHz.

In order to test the short repeatability of measured respiratory modulation frequency, the root mean square (RMS) difference between repeats was calculated for OscP and KorS.

#### Respiratory amplitude modulation

The respiratory amplitude modulation of OscP and KorS was defined as the peak-to-nadir difference normalized to the mean amplitude over the 20 s analysis period.

#### Phase shift

The phase shifts between Resp and the modulated amplitudes of OscP and KorS were calculated as the phase difference between the first peak of the cross-correlation function and the zero-lag point. Similarly, the phase difference between the amplitudes of OscP and KorS was calculated.

### Statistical analysis

The effect of respiratory rate and cuff pressure on KorS and OscP amplitude, phase shift, RR interval and respiratory depth was studied by three-way analysis of variance (ANOVA) using MATLAB Statistical Toolbox™ software (The Mathworks, Natick, MA, USA). Post hoc multiple comparison was done using the two-tail *t* test for paired samples. A significance level *α* = 0.05 was adopted.

## Results

### Presence of respiratory modulation of oscillometric pulses and Korotkoff sounds

**Fig. 2:**
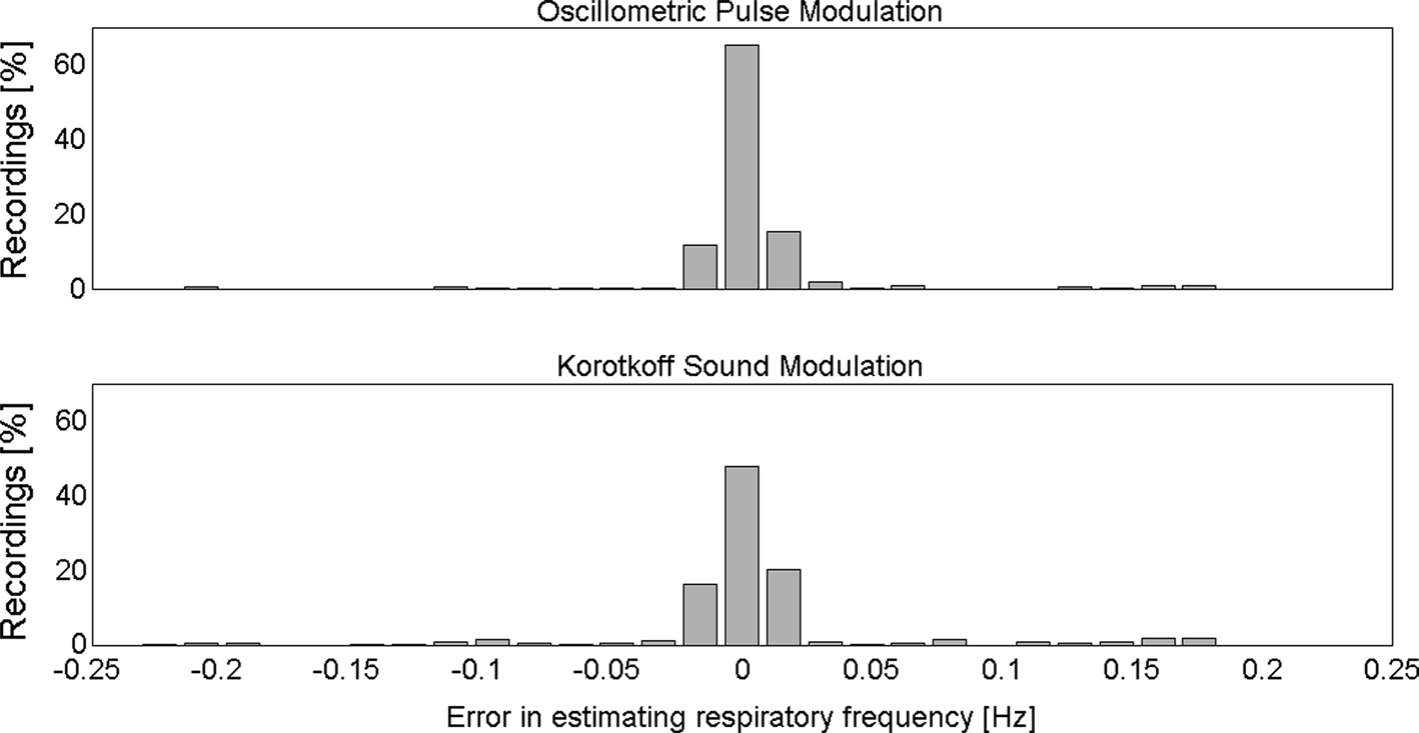
Distribution of respiratory rate estimation error across all recordings (*N* = 360). Oscillometric pulse amplitude modulation (*top panel*) and Korotkoff sound amplitude modulation (*bottom*). Bin width (the same as the spectral resolution) is 16 mHz

As shown in Fig. 2, there was no statistically significant difference in respiratory rate from Resp and that estimated from either OscP amplitude or KorS amplitude, indicating that these signals were modulated by respiration. Table 2 shows the number of recordings with respiratory modulation. Respiratory modulation of OscP and KorS amplitudes was observed in all 30 subjects. For OscP amplitude, modulation was observed in all subjects in eight or more recordings, and in 17 subjects modulation was observed in all recordings. For KorS amplitude, they were six or more recordings and nine subjects, respectively.

**Table 2:** Estimation of respiratory rate

	All recordings			Recordings with respiratory modulation	
	Number	RMS difference (mHz)		Number (%)	
		OscP	KorS	OscP	KorS
All	360	8	10	332 (92 %)	304 (84 %)
Resp 0.2 Hz	180	10	11	165 (92 %)	156 (87 %)
Resp 0.3 Hz	180	7	9	167 (93 %)	148 (82 %)
Cuff H	120	9	11	115 (96 %)	104 (87 %)
Cuff M	120	8	9	116 (97 %)	99 (83 %)
Cuff L	120	8	11	101 (84 %)	101 (84 %)

Cuff H, M, L are for cuff pressures high, medium, low

The presence of respiratory modulation was accepted when the modulated peak frequency in OscP or KorS amplitude was detected within the spectral resolution of 16 mHz

There was also no statistically significant difference in measured respiratory modulation frequency between repeated recordings for either OscP or KorS. The RMS difference between repeats was 10 mHz for OscP and 11 mHz for KorS.

### Respiratory modulation of amplitude for oscillometric pulses and Korotkoff sounds

**Fig. 3:**
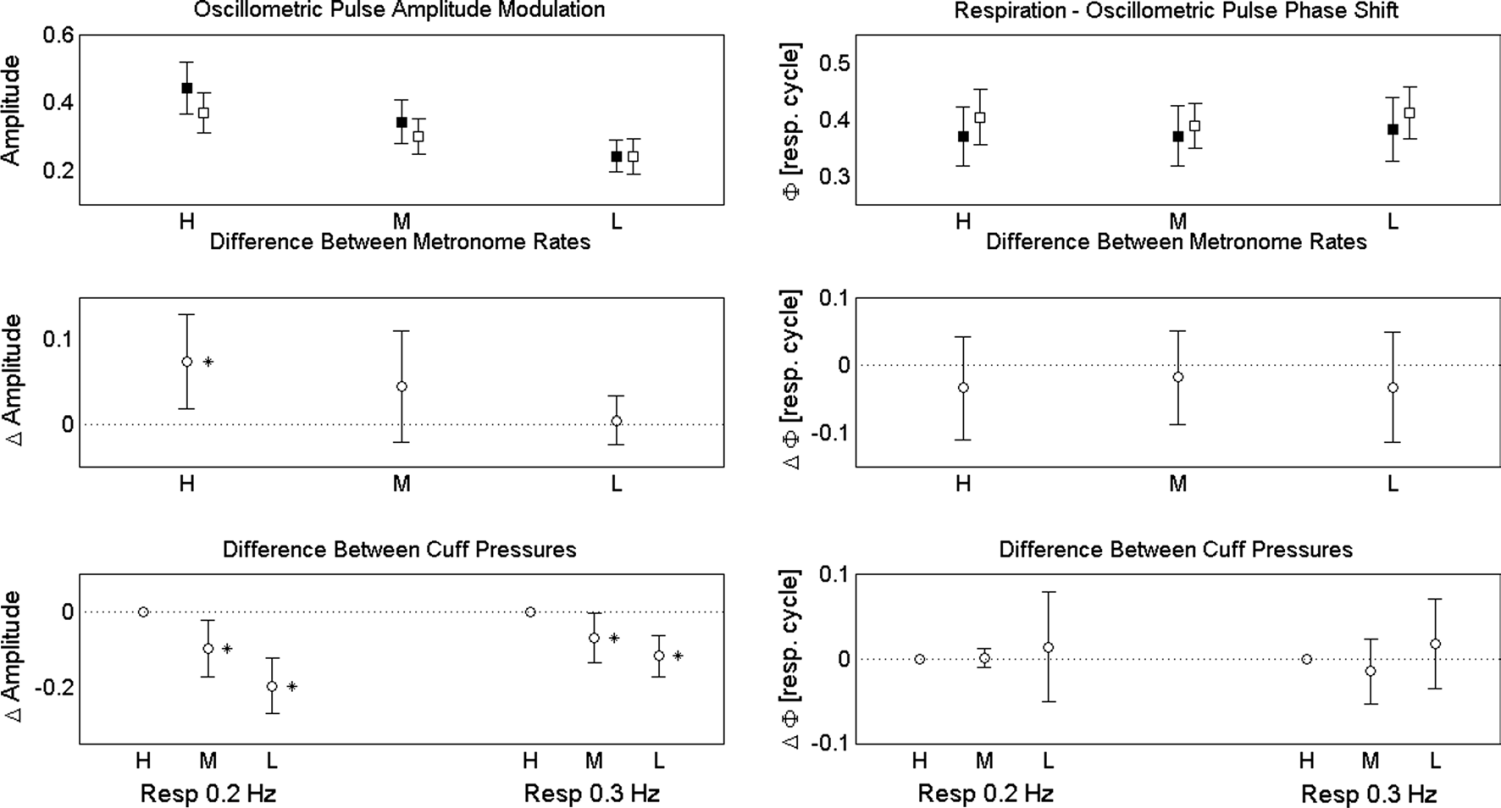
*Top panels* oscillometric amplitude modulation (*left*) and phase shift from respiratory magnetometer (*right*) with two respiratory rates (*black square* for 0.2 Hz, white square for 0.3 Hz) and three cuff pressures (H, M and L). The mean values and 95 % confidence intervals are given. *Central panels* comparison of oscillometric amplitude modulation (*left*) and phase shift from respiratory magnetometer (*right*) between the two respiratory rates (referenced to the values from 0.3 Hz). *Bottom panels* comparison of oscillometric amplitude modulation (*left*) and phase shift from respiratory magnetometer (*right*) between the three cuff pressures [referenced to high cuff pressure (H)]. The mean difference and 95 % confidence intervals of difference are given. *Asterisk* indicates *p* < 0.05

OscP amplitude modulation changed significantly between the two respiratory rates (with a mean and SD of the difference of 0.04 ± 0.09, *p* < 0.05) and also between the three cuff pressures (*p* < 0.0001), whereas no statistically significant changes were seen with KorS amplitude (Table 3). The statistically significant changes for OscP modulation with different cuff pressures showed a decrease with decreasing cuff pressure, which was significantly reduced at both the medium (M) and low (L) cuff pressures (*p* < 0.05) (Fig. 3), with a mean and SD of difference of −0.08 ± 0.14 and −0.16 ± 0.15 when referenced to the high (H) cuff pressure.

**Table 3:** Oscillometric pulse (OscP) and Korotkoff sound (KorS) amplitude modulation at different respiratory rates and cuff pressures

	Respiratory rate		*p* value	Cuff pressure			*p* value
	0.2 Hz	0.3 Hz		H	M	L	
OscP	0.34 ± 0.13	0.30 ± 0.12	<0.05	0.40 ± 0.17	0.32 ± 0.13	0.24 ± 0.12	<0.0001
KorS	1.87 ± 0.92	1.65 ± 0.72	N.S.	1.91 ± 1.12	1.58 ± 0.72	1.75 ± 1.19	N.S.

Data are presented as mean ± SD

Amplitude modulation is defined as the change from minimum to maximum divided by the mean amplitude over the 20-s analysis period

### Respiratory modulation of phase relationships for oscillometric pulses and Korotkoff sounds

The phase shift between Resp and the OscP amplitude modulation was statistically significant between the two respiratory rates (with a mean and SD of the difference of 0.03 ± 0.09 cycle, *p* < 0.05) (Fig. 3 and Table 4). However, the difference was not statistically significant for different cuff pressures (Table 4). The phase difference between OscP and KorS amplitudes was also not statistically significant for either respiratory rate or cuff pressure (Table 4).

**Table 4:** Phase shift changes with respiratory rate and cuff pressure

	Respiratory rate		*p* value	Cuff pressure			*p* value
	0.2 Hz	0.3 Hz		H	M	L	
Resp–OscP	0.37 ± 0.12	0.40 ± 0.09	<0.05	0.38 ± 0.11	0.39 ± 0.15	0.39 ± 0.11	N.S.
Resp–KorS	0.37 ± 0.12	0.40 ± 0.10	N.S.	0.39 ± 0.09	0.38 ± 0.08	0.40 ± 0.08	N.S.
OscP–KorS	0.00 ± 0.08	−0.01 ± 0.09	N.S.	0.00 ± 0.05	−0.01 ± 0.11	0.00 ± 0.13	N.S.

Data are presented as mean ± SD

Phase shift is given as a fraction of the respiratory cycle. Positive values indicate delay of the second signal with respect to the first

### Heart rate and respiratory depth changes with respiratory frequency

There was no statistically significant difference in RR interval (mean ± SD of 0.88 ± 0.12 s for all the conditions) between the two respiratory rates and between the three cuff pressures (*p* > 0.05). Respiratory depth significantly increased when breathing at the slower rate (0.2 Hz) compared with the higher rate (0.3 Hz) by 25 ± 10 % (*p* < 0.0001). This is as expected physiologically as respiratory depth needs to increase with slower respiration to allow transfer of similar oxygen levels.

## Discussion

This study has shown significant respiratory modulation of the oscillometric pulse and Korotkoff sound amplitudes from which BP is measured. If the amplitude of KorS and OscP signals was modulated by respiration, the respiratory frequency calculated from KorS and OscP would be expected to be the same at that from Resp. Our results of the nonsignificant difference of respiratory rate from Resp and that estimated from either OscP amplitude or KorS amplitude confirmed this hypothesis. In this study, respiratory modulation was discovered in all subjects, but in a higher proportion of recordings for oscillometric pulses (92 % compared with 84 %). This could have been caused by poorer signal quality of the recorded Korotkoff sound, perhaps due to greater depth of the brachial artery in some subjects or physiological properties of the arterial wall, or due to poor contact between the stethoscope and skin, or higher sensitivity to slight movements of the arm during measurement.

The respiratory depth was characterized by significantly larger amplitude at the lower respiratory rate (0.2 Hz, 12 breaths/min), and this was associated with higher OscP amplitude modulation. This result supports the hypothesis of a direct mechanical coupling between respiration and the vasculature suggested by Saul et al. [13].

The respiratory modulation of OscP amplitude decreased with cuff pressure at both respiratory rates (Fig. 3), suggesting mechanical coupling delivers higher energy at increased load (resistance to arterial vessel distension occurring at higher cuff pressures). This hypothesis seems to be confirmed by the significantly higher OscP amplitude modulation at the high cuff pressure, for the lower respiratory rate which is associated with larger respiratory movement of the chest.

A statistically significant larger phase shift was observed between the respiratory signal recorded by the chest magnetometer and the oscillometric pulse amplitude modulation. Sin and colleagues [14] studied the phase relationship between respiratory depth and the SBP envelope. In their Fig. 6, the lower left panel shows a phase delay of 0.2 of a respiratory cycle for the SBP maximum with respect to the beginning of inspiration for a respiratory rate of 0.2 Hz. Assuming a regular respiratory pattern, 0.25 cycles are required to reach the inspiration peak, giving a total of 0.35 cycles delay between respiratory peak and SBP maximum. This result was consistent with ours for the same respiratory rate (Table 4).

Sin et al. [14] also examined lower respiratory rates, down to 0.1 Hz and found a decreasing trend in phase delay with increasing respiratory rate, which suggests that a larger tidal volume (lower respiratory rate) is associated with increased inertial delay in the mechanical coupling between respiration and the arterial hemodynamic response. However, they did not analyze respiratory rates higher than 0.2 Hz. In our study, for a respiratory rate of 0.3 Hz, we found a significantly increase in phase delay with respect to the lower rate of 0.2 Hz; namely, an inversion of the trend observed by Sin and colleagues for lower rates up to 0.2 Hz. This could be caused by the effects of non-mechanical processes such as hyperventilation that come into play only at higher respiratory rates.

Our results also appeared consistent with the phase shift analysis of Saul et al. [13]. Although numeric values were not reported in their study, their Fig. 3 showed a between-subject mean phase delay of the pulse pressure with respect to the respiratory signal (instantaneous lung volume) of approximately −110°, or 31 % of the respiratory cycle, which was similar for the respiratory rates of 0.2 and 0.3 Hz. However, their figure illustrated only group means for each frequency, and therefore, it is uncertain whether the phase shift varied significantly with the respiratory rate.

One limitation of this research was that only 30 healthy subjects with normal BPs were studied, and the results, although providing physiological insight, were based on statistical significance and not clinical significance. A future clinical study with a large sample size, including both hypotensive and hypertensive subjects, would be a useful investigation of the potential effect of disease and would help determine clinical significance. However, this work has already made an important step in understanding the modulation effect of respiration on oscillometric pulses and Korotkoff sounds. An important clinical implication is that amplitude changes for oscillometric pulses and Korotkoff sounds are commonly used for BP determination; and hence understanding that additional amplitude changes caused by respiration could help improve the accuracy of clinical BP measurement by advising patients to limit deeper breathing during BP measurement. Also of relevance is that the respiratory rate can be derived from the oscillometric pulse and Korotkoff sound amplitude. This would add an additional physiological parameter during normal BP measurement.

In conclusion, this study has shown quantitative evidence of a respiratory modulation of the oscillometric pulse and Korotkoff sound amplitude in noninvasive BP measurement in healthy normotensive subjects, implicating the influence of respiration on clinical BP measurement.
